# Prognostic Value of Estimated Glomerular Filtration Rate in Older Patients With Acute Coronary Syndrome

**DOI:** 10.31083/RCM45446

**Published:** 2026-02-11

**Authors:** Yifan Li, Tiantian Sang, Zuozhi Li, Naqiang Lv, Jinxing Liu, Yingzhen Gu, Xiaorong Han, Wei Zhang, Aimin Dang

**Affiliations:** ^1^Department of Cardiology, Fuwai Hospital, National Center for Cardiovascular Diseases, Chinese Academy of Medical Sciences and Peking Union Medical College, 100037 Beijing, China; ^2^Department of Geriatrics, Beijing Jishuitan Hospital, Capital Medical University, 100035 Beijing, China

**Keywords:** glomerular filtration rate, aged, 80 and over, acute coronary syndrome, prognosis

## Abstract

**Background:**

While the association between estimated glomerular filtration rate (eGFR) and cardiovascular disease has been well established in younger populations, the prognostic significance of this marker in older individuals remains less well defined. Thus, this study aimed to evaluate the predictive value of eGFR in patients aged 80 years or older with acute coronary syndrome (ACS).

**Methods:**

We enrolled 551 patients aged ≥80 years hospitalized for ACS, who had the eGFR calculated at admission. The participants were further stratified into three groups by eGFR levels: Low-eGFR group (L-eGFR, eGFR < the 20th percentile), Medium-eGFR group (M-eGFR, the 20th percentile ≤ eGFR < the 80th percentile), and High-eGFR group (H-eGFR, eGFR ≥ the 80th percentile). Major adverse cardiovascular events (MACEs) were recorded during the follow-up period.

**Results:**

During a median 63-month follow-up, the L-eGFR group exhibited a higher cumulative incidence of MACEs, while the H-eGFR group showed a relatively improved prognosis compared with the M-eGFR group. A multivariate Cox regression analysis revealed that reduced eGFR levels remained independently predictive for long-term MACEs. Compared with the M-eGFR group, the L-eGFR group showed a higher risk (hazard ratio (HR) 1.542, 95% confidence interval (CI): 1.104–2.155). The H-eGFR group exhibited a protective effect (HR 0.643, 95% CI: 0.438–0.943).

**Conclusions:**

Reduced eGFR levels were independent predictors for long-term MACEs in older ACS patients. The H-eGFR group had an improved prognosis, suggesting that further exploration of the underlying mechanism linking renal function and prognosis is warranted.

## 1. Introduction

Coronary artery disease (CAD) stands as a predominant contributor to global morbidity and mortality, representing a critical public health challenge [1, 2]. Acute coronary syndrome (ACS) is a serious type of CAD with a poor prognosis, especially in the elderly [3]. Despite the promotion of healthy lifestyles, controlling cardiovascular risk factors and appropriate antithrombotic treatments, ischemic events still occur [4]. This fact suggests that targeting traditional risk factors may not be sufficient to improve clinical outcomes, and new targets and therapies need to be identified.

Chronic kidney disease (CKD) imposes a growing burden on public health and is closely linked to cardiovascular disease (CVD) [5, 6]. Estimated glomerular filtration rate (eGFR) serves as a common clinical indicator for renal function. Several studies have shown that lower eGFR was associated with an increased cardiovascular risk [7, 8, 9, 10, 11]. However, emerging evidence points to a U-shaped relationship between eGFR and mortality, suggesting that renal hyperfiltration might be a powerful predictor for poor cardiovascular prognosis [12, 13].

The World Health Organization (WHO) defines the population aged 80 and above as “oldest-old”, who are rapidly increasing worldwide and have different physiological status from younger populations due to aging, frailty, malnutrition and comorbidities [14]. Advanced age is associated with a rising prevalence of chronic conditions such as hypertension and diabetes. The cumulative effect of these diseases and their complications significantly diminishes the quality of life and increases the susceptibility to adverse clinical outcomes in older individuals. Frailty is a common geriatric syndrome reflecting decreased physiological reserve and increased vulnerability to stressors [15] and poses a higher risk of readmission and mortality [16]. A community-based study in China reported an overall frailty prevalence of 9.9% in the elderly, which rose sharply to 26% among those aged 80 and above [17]. Despite growing research interest in the elderly, advanced-age patients are still systematically excluded from large clinical trials. Less than 10% of ACS trials include patients over 75 [18], leading to limited generalizability of findings in this population. To address this gap, the present study aimed to determine the prognostic role of baseline eGFR in ACS patients aged 80 years and older.

## 2. Materials and Methods

### 2.1 Study Population

**Fig. 1.**
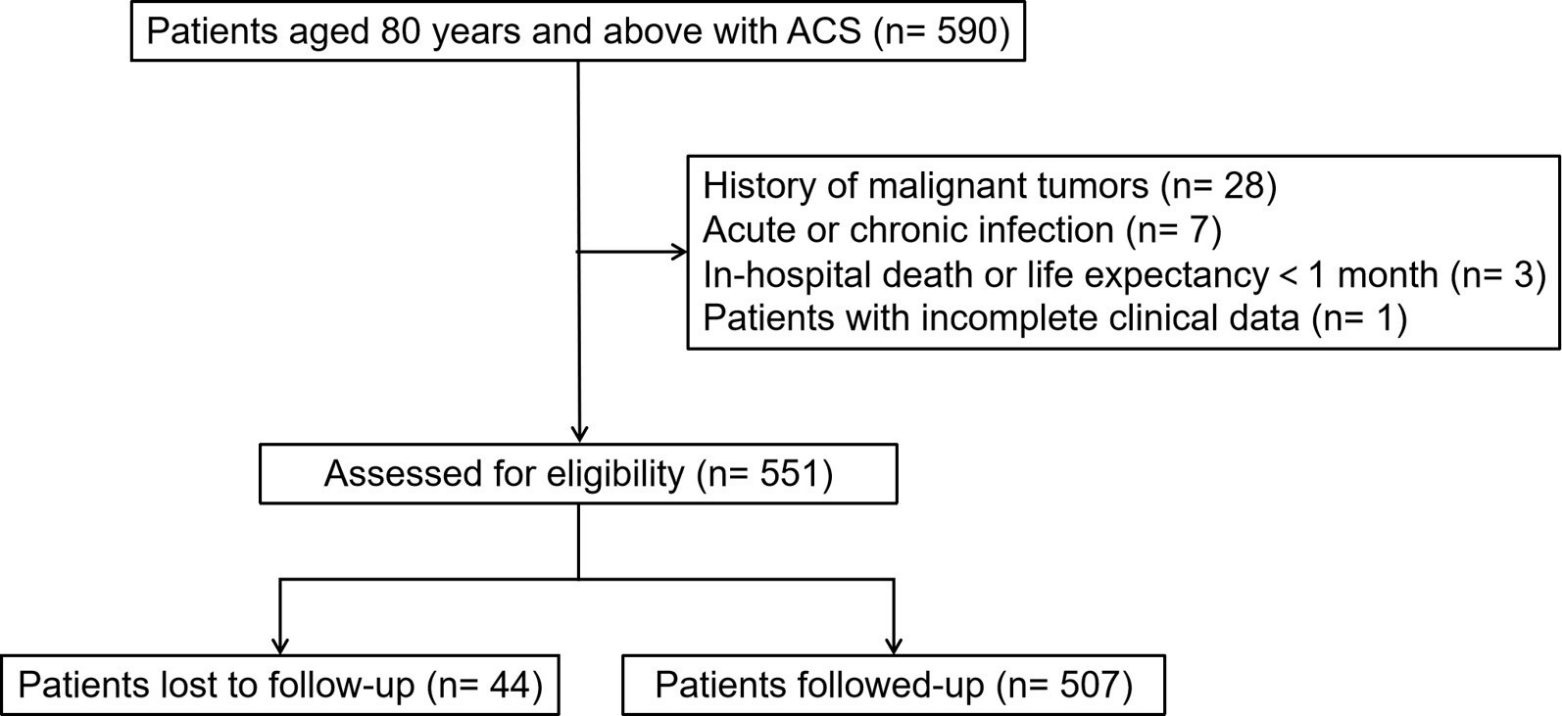
**Flow diagram of patient selection**. ACS, acute coronary syndrome.

This study included patients aged 80 years or above with a primary diagnosis of ACS, who were admitted to Fuwai Hospital (Beijing, China) between January 2011 and February 2016. The exclusion criteria were: history of malignant tumors, acute or chronic infection, in-hospital death or life expectancy less than 1 month after discharge, and patients with incomplete data. The final study population comprised 551 individuals, of whom 44 were lost to follow-up (Fig. 1).

According to the eGFR levels calculated at admission, the participants were further divided into three groups: Low-eGFR group (L-eGFR, eGFR < the 20th percentile), Medium-eGFR group (M-eGFR, the 20th percentile ≤ eGFR < the 80th percentile) and High-eGFR group (H-eGFR, eGFR ≥ the 80th percentile). Considering the number of individuals included and the methods applied in previous studies [19], we used the L-eGFR group and the H-eGFR group as representatives for renal hypofiltration and hyperfiltration. This study conformed to the Declaration of Helsinki, and was approved by the Medical Ethics Committee of Fuwai Hospital [Approval Number: 2021-1461]. Written informed consent was formally waived.

### 2.2 Data Collection and Definitions

The demographic and clinical data was collected through electronic medical records by trained physicians. The data included gender, age, height, weight, cardiovascular risk factors, medical history, and vital signs measured at admission. Fasting venous blood samples were obtained from all enrolled participants. All laboratory assays were subsequently performed by the clinical chemistry department of the Fuwai Hospital.

Definitions for related diseases and conditions were as follows: (1) Hypertension: systolic blood pressure ≥140 mmHg or diastolic blood pressure ≥90 mmHg based on the average of three separate measurements, previous diagnosis by physician, or current use of antihypertensive medication. (2) Diabetes mellitus: fasting plasma glucose ≥7.0 mmol/L, 2-hour postprandial glucose ≥11.1 mmol/L in oral glucose tolerance test, random plasma glucose ≥11.1 mmol/L, previous diagnosis by physician, or current use of insulin or hypoglycemic medication. (3) Dyslipidemia: triglycerides ≥1.7 mmol/L, total cholesterol ≥5.2 mmol/L, high-density lipoprotein cholesterol <1.0 mmol/L, low-density lipoprotein cholesterol ≥3.4 mmol/L, previous diagnosis by physician, or current use of lipid-lowering medication. (4) Smoking status included both current smokers and former smokers. (5) History of myocardial infarction, atrial fibrillation, or stroke was obtained from prior medical records.

Body mass index (BMI) was calculated as the ratio of weight to height squared (kg/m^2^). The eGFR was calculated using the Modification of Diet in Renal Disease (MDRD) formula: eGFR (mL/min per 1.73 m^2^) = 186 × serum creatinine (mg/dL)^-1.154^ × age^-0.203^ × 0.742 (if female) × 1.233.

### 2.3 Follow-up and Outcomes

Follow-up was performed by trained physicians via clinic visits or phone calls in September 2020. The endpoint of this study was major adverse cardiovascular events (MACE), including all-cause mortality, non-fatal myocardial infarction, unplanned target vessel revascularization, non-fatal stroke/transient ischemic attack (TIA), and readmission due to heart failure.

### 2.4 Statistical Analysis

Continuous variables were tested for normal distribution with the Kolmogorov-Smirnov test. Based on their distribution, they were expressed as mean ± standard deviation or median (interquartile range). Student’s *t*-test or Mann-Whitney U test was used between two groups, while ANOVA, Welch test, or Kruskal-Wallis H test was applied among multiple groups for comparison. Categorical variables, expressed as frequency (percentage), were compared using the Chi-square test. Survival analysis involved plotting Kaplan-Meier curves and performing log-rank tests. Restricted cubic splines (RCS) were employed to assess nonlinear relationship between eGFR and MACE. Then, we established univariate and multivariate Cox regression models to explore the prognostic impact of eGFR. Interaction effects were investigated through subgroup analyses. Sensitivity analysis was used to enhance the robustness of the findings. All analysis was conducted in R version 4.3.2 (R Foundation for Statistical Computing, Vienna, Austria), with a two-tailed *p* < 0.05 indicating significance.

## 3. Results

### 3.1 Baseline Characteristics

A total of 551 patients were enrolled, with a median age of 81 years and a male predominance of 62.1%. In this cohort, hypertension and dyslipidemia were prevalent in nearly 80%, while diabetes mellitus was present in more than one-third.

Table 1 summarizes the baseline characteristics of the study population, which were classified into three groups: the L-eGFR group (eGFR <68.00 mL/min per 1.73 m^2^), the M-eGFR group (68.00 ≤ eGFR < 108.00 mL/min per 1.73 m^2^), and the H-eGFR group (eGFR ≥108.00 mL/min per 1.73 m^2^). Compared with the M-eGFR group and H-eGFR group, patients in L-eGFR group had a higher prevalence of prior myocardial infarction, higher levels of uric acid and inflammatory indicators, and poorer cardiac function. Significant differences were absent among the three groups regarding traditional risk factors.

**Table 1.** **Baseline characteristics of study population**.

Variables		Overall (N = 551)	L-eGFR (N = 111)	M-eGFR (N = 330)	H-eGFR (N = 110)	*p* value
Male, n (%)		342 (62.1%)	49 (44.1%)*	219 (66.4%)	74 (67.3%)	<0.001
Age, years		81.00 (80.00, 83.00)	82.00 (80.00, 83.00)	81.00 (80.00, 83.00)	81.00 (80.00, 82.00)	0.018
BMI, kg/m^2^		24.4 ± 3.3	24.9 ± 3.4	24.4 ± 3.2	23.8 ± 3.2	0.173
Hypertension, n (%)		438 (79.5%)	96 (86.5%)	257 (77.9%)	85 (77.3%)	0.123
Dyslipidemia, n (%)		441 (80.0%)	97 (87.4%)	259 (78.5%)	85 (77.3%)	0.092
Diabetes, n (%)		194 (35.2%)	46 (41.4%)	106 (32.1%)	42 (38.2%)	0.158
Smoking, n (%)		229 (41.6%)	38 (34.2%)	148 (44.8%)	43 (39.1%)	0.123
Prior MI, n (%)		128 (23.2%)	37 (33.3%)*	73 (22.1%)	18 (16.4%)	0.009
History of stroke/TIA, n (%)		140 (25.4%)	29 (26.1%)	79 (23.9%)	32 (29.1%)	0.551
Atrial fibrillation, n (%)		92 (16.7%)	28 (25.2%)	58 (17.6%)	6 (5.5%)	<0.001
Classification of ACS						0.043
	Unstable angina, n (%)	381 (69.1%)	65 (58.6%)	238 (72.1%)	78 (70.9%)	
	NSTEMI, n (%)	85 (15.4%)	20 (18.0%)	51 (15.5%)	14 (12.7%)	
	STEMI, n (%)	85 (15.4%)	26 (23.4%)	41 (12.4%)	18 (16.4%)	
HR, bpm		67 (62, 75)	68 (62, 76)	67 (62, 75)	68 (61, 77)	0.974
SBP, mmHg		130 (120, 140)	130 (120, 141)	130 (120, 141)	130 (120, 140)	0.088
DBP, mmHg		70 (63, 80)	70 (60, 79)	70 (63, 80)	70 (67, 80)	0.057
Leukocyte, ×10^9^/L		6.49 (5.46, 7.83)	7.28 (6.08, 8.53)	6.45 (5.44, 7.79)	5.96 (5.03, 7.09)	<0.001
Albumin, g/L		39.4 (36.7, 42.5)	39.2 (36.7, 42.0)	39.7 (36.7, 42.9)	39.1 (36.8, 42.5)	0.556
TG, mmol/L		1.28 (0.94, 1.71)	1.50 (1.13, 2.07)*	1.26 (0.95, 1.67)	1.09 (0.88, 1.48)	<0.001
TC, mmol/L		3.84 (3.31, 4.46)	3.99 (3.49, 4.59)	3.82 (3.30, 4.38)	3.68 (3.13, 4.38)	0.086
HDL-C, mmol/L		1.08 (0.91, 1.30)	1.05 (0.87, 1.24)	1.10 (0.92, 1.30)	1.08 (0.93, 1.35)	0.100
LDL-C, mmol/L		2.23 (1.80, 2.69)	2.29 (1.78, 2.82)	2.23 (1.80, 2.68)	2.09 (1.67, 2.60)	0.182
eGFR, mL/min per 1.73 m^2^		88.98 ± 24.88	56.28 ± 10.59*	87.88 ± 11.12*	125.29 ± 14.63*	<0.001
Uric acid, µmol/L		345.14 (280.56, 414.28)	427.25 (344.07, 506.90)*	346.42 (291.89, 406.65)*	273.59 (242.86, 344.02)*	<0.001
hsCRP, mg/L		2.37 (1.20, 7.39)	4.21 (1.54, 11.12)	2.21 (1.05, 6.19)	1.93 (1.22, 4.60)	<0.001
HbA1c, %		6.2 (5.8, 6.8)	6.4 (5.9, 7.5)	6.2 (5.8, 6.7)	6.2 (5.9, 6.9)	0.257
NT-proBNP, pg/mL		692.8 (366.2, 1230.2)	1016.1 (500.6, 2145.0)*	680.2 (371.0, 1200.9)	601.4 (270.1, 845.5)	<0.001
LVEF, %		60.0 (56.0, 65.0)	58.6 (52.0, 62.0)*	60.4 (56.6, 65.0)	60.1 (56.0, 65.0)	<0.001

Abbreviations: ACS, acute coronary syndrome; BMI, body mass index; DBP, diastolic blood pressure; eGFR, estimated glomerular filtration rate; L-eGFR, Low-eGFR; M-eGFR, Medium-eGFR; H-eGFR, High-eGFR; HbA1c, hemoglobin A1c; HDL-C, high-density lipoprotein cholesterol; HR, heart rate; hsCRP, high-sensitivity C-reactive protein; LDL-C, low-density lipoprotein cholesterol; LVEF, left ventricular ejection fraction; MI, myocardial infarction; NSTEMI, non-ST-segment elevation myocardial infarction; NT-proBNP, N-terminal pro-brain natriuretic peptide; SBP, systolic blood pressure; STEMI, ST-segment elevation myocardial infarction; TC, total cholesterol; TG, triglyceride; TIA, transient ischemic attack. The asterisk “*” indicates statistical significance compared to the other two groups based on post-hoc pairwise comparisons with Bonferroni correction.

### 3.2 Patient Outcomes

Over a median follow-up of 63 (44–79) months, 44 patients were lost to follow-up. There were no significant differences in demographic characteristics, traditional cardiovascular risk factors, or renal function between the follow-up and lost populations (**Supplementary Table 1**). Out of 507 follow-up patients, 247 individuals developed MACE, who had a higher incidence of smoking, prior myocardial infarction, atrial fibrillation, and previous stroke/TIA. The patients in the MACE group had decreased renal function (**Supplementary Table 2**).

**Fig. 2.**
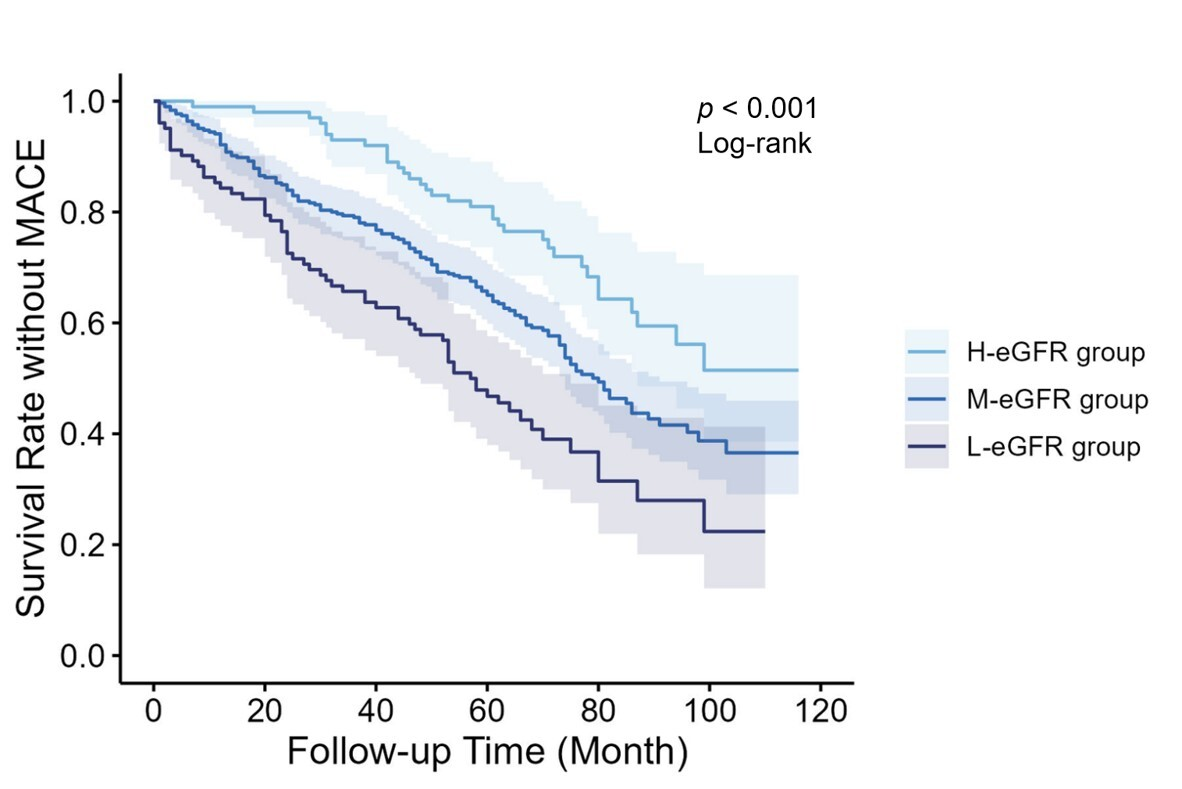
**Kaplan Meier survival curves for different groups**. eGFR, estimated glomerular filtration rate; MACE, major adverse cardiovascular events.

As shown in Fig. 2, Kaplan Meier curves indicated that long-term clinical outcomes varied among groups (Log-rank *p* < 0.001). Compared with the M-eGFR group, patients in the L-eGFR group had a higher cumulative incidence of MACE, while H-eGFR group patients showed a better prognosis.

### 3.3 Predictive Value of eGFR

After adjustment in several models in RCS, eGFR did not show a nonlinear correlation with long-term MACE (**Supplementary Fig. 1**). For a more intuitive clinical risk stratification, the eGFR was converted into a categorical variable for subsequent analysis.

Cox regression analysis models were established to explore predictors for MACE (Table 2). Univariate Cox analysis showed that age, higher uric acid levels, reduced eGFR levels and left ventricular ejection fraction (LVEF) were associated with an increased risk of long-term MACE. After adjustment for gender, age, BMI, albumin and uric acid levels, lower eGFR persisted as an independent risk factor. Compared with the M-eGFR group, L-eGFR was an independent predictor for long-term MACE (hazard ratio (HR) 1.542, 95% confidence interval (CI): 1.104–2.155, *p* = 0.011). Conversely, H-eGFR showed a protective effect (HR 0.643, 95% CI: 0.438–0.943, *p* = 0.024).

**Table 2.** **Predictors for MACE in univariate and multivariate Cox regression analyses**.

Variables		HR	95% CI	*p* value
		Univariate		
Age		1.074	(1.017–1.134)	0.010
Gender		1.052	(0.812–1.363)	0.700
BMI		1.013	(0.974–1.054)	0.510
Hypertension		1.093	(0.789–1.514)	0.593
Diabetes mellitus		1.264	(0.979–1.632)	0.072
Smoke		1.252	(0.974–1.609)	0.080
HR		1.008	(0.996–1.019)	0.197
SBP		1.005	(0.999–1.012)	0.110
Albumin		0.975	(0.946–1.004)	0.095
HDL-C		0.701	(0.463–1.060)	0.092
LDL-C		0.996	(0.849–1.169)	0.963
Uric acid		1.003	(1.001–1.004)	<0.001
eGFR		0.987	(0.982–0.993)	<0.001
HbA1c		1.090	(0.972–1.223)	0.141
LVEF		0.964	(0.950–0.977)	<0.001
M-eGFR group		Reference		
L-eGFR group		1.729	(1.287–2.322)	<0.001
H-eGFR group		0.579	(0.399–0.840)	0.004
		Multivariate		
Model 1				
	eGFR	0.988	(0.982–0.993)	<0.001
	M-eGFR group	Reference		
	L-eGFR group	1.767	(1.304–2.396)	<0.001
	H-eGFR group	0.601	(0.413–0.876)	0.008
Model 2				
	eGFR	0.991	(0.985–0.997)	0.006
	M-eGFR group	Reference		
	L-eGFR group	1.542	(1.104–2.155)	0.011
	H-eGFR group	0.643	(0.438–0.943)	0.024

Model 1: adjusted for age, gender and BMI; Model 2: adjusted for model 1 plus albumin and uric acid. HR, hazard ratio; CI, confidence interval.

### 3.4 Subgroup Analysis

**Fig. 3.**
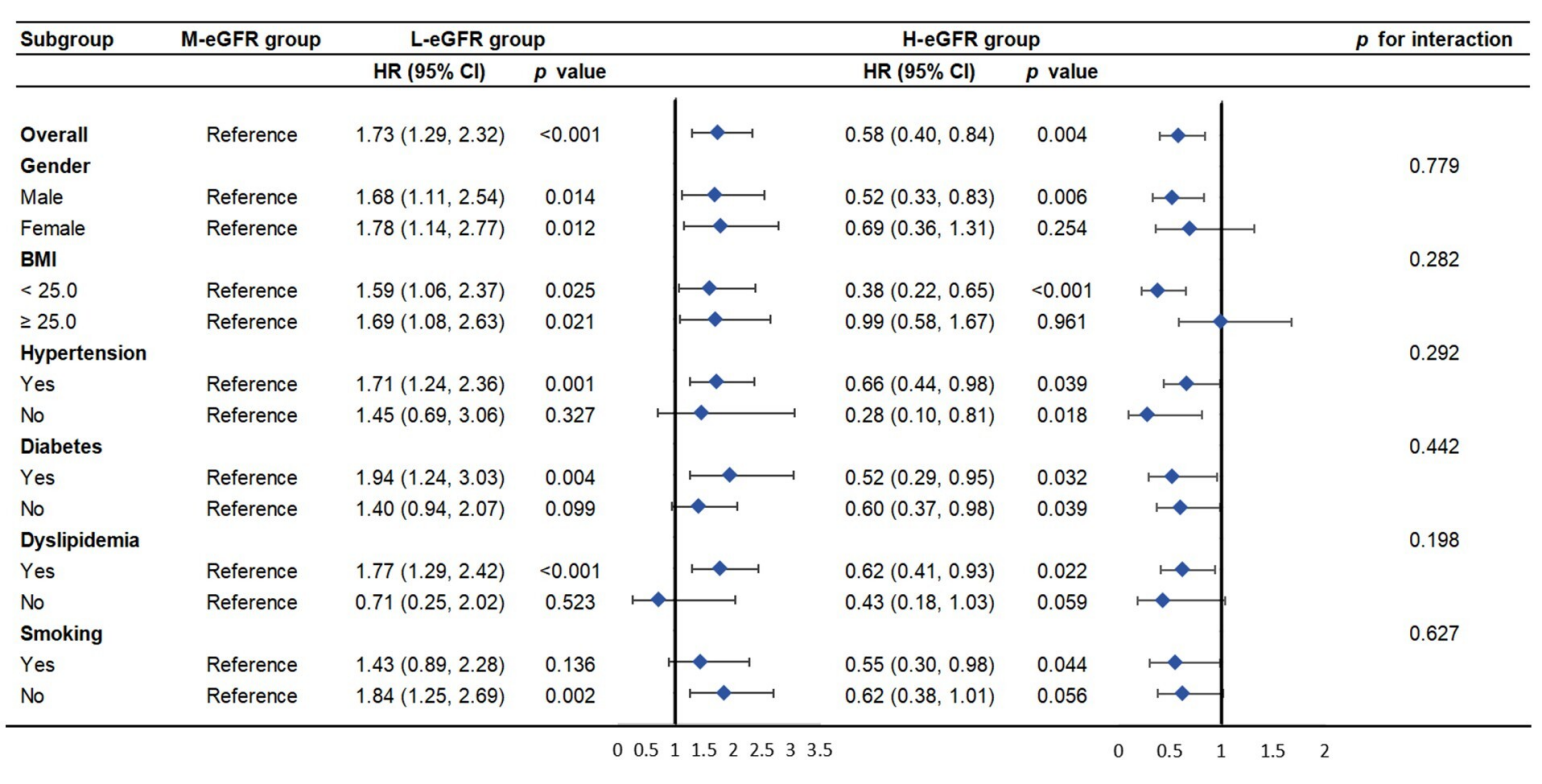
**Subgroup analysis of the association between eGFR and MACE among older participants with ACS**.

Individuals were grouped based on gender, BMI (<25 or ≥25), hypertension, diabetes, dyslipidemia, and smoking status in the subgroup analysis. No statistically significant interaction effect was observed (both *p* for interaction >0.05) (Fig. 3).

### 3.5 Sensitivity Analysis

Sensitivity analysis was performed using two distinct eGFR categorizations. First, patients were stratified by eGFR tertiles (T1 group: <77.03; T2 group: 77.03–98.88; T3 group: ≥98.88 mL/min per 1.73 m^2^). Kaplan Meier curves demonstrated a worse prognosis in the T1 group compared with the T2 and T3 group, with no statistically significant difference observed between the latter two (**Supplementary Fig. 2**). In the Cox regression analysis using the T2 group as the reference, the T1 group remained an independent risk factor for MACE, whereas the T3 group showed no significant association with long-term MACE (**Supplementary Table 3**). Second, patients were categorized according to CKD staging criteria (group 1: <60.00; group 2: 60.00–90.00; group 3: ≥90.00 mL/min per 1.73 m^2^). Kaplan Meier curves yielded results consistent with the primary analysis (**Supplementary Fig. 3**). However, in the multivariate Cox model with group 2 as the reference, group 3 exhibited a protective effect, while group 1 did not show independent prognostic value (**Supplementary Table 4**).

These analyses support a linear trend between eGFR and MACE, and further suggest a potential plateau in the protective effect at higher eGFR levels, which is also reflected in the RCS analysis.

## 4. Discussion

Advancing age is an important predictor of adverse outcomes following ACS [20]. However, large-scale randomized controlled trials mostly excluded the elderly population ≥80 years of age, which has dramatically increased in the past decades. To address this gap, we conducted what is, to our knowledge, the first study systematically designed to explore the prognostic value of eGFR and potential role of renal hyperfiltration in ACS patients aged 80 and above. The baseline data showed that the majority of participants had experienced hypertension, diabetes mellitus, dyslipidemia, and other cardiovascular risk factors. Survival analysis revealed that reduced eGFR was an independent predictor of the long-term risk for MACE. Notably, while L-eGFR was associated with an increased risk for MACE, patients in the H-eGFR group exhibited better clinical outcomes compared to those in the M-eGFR group.

In recent years, eGFR has emerged as a predictor of cardiovascular risk in different populations. Based on the data of the UK Biobank, Lees *et al*. [21] reported that the incidence of cardiovascular events gradually increased with the decline of eGFR. A Chinese cohort study of 28,187 individuals yielded similar conclusions [22]. In patients with ACS, reduced eGFR predicts not only the risk of in-hospital death [23], but also adverse long-term outcomes [24, 25]. Nevertheless, there is limited data supporting eGFR as a predictor of cardiovascular outcome in older populations, especially those over 80 years. Our study, including more than 550 ACS patients aged ≥80 years, demonstrated that the decrease in eGFR was an independent risk factor for long-term MACE. Patients with reduced eGFR often present with comorbidities such as hypertension, diabetes, and dyslipidemia, which promote the development of atherosclerosis through inflammatory and oxidative pathways. In addition, CKD patients usually exhibit chronic inflammation and dysregulation of calcium-phosphorus metabolism. Uremic toxin-related endothelial damage also accelerates the process of atherosclerosis and myocardial remodeling [26]. In summary, this study reached similar conclusions to previous studies regarding the impact of reduced eGFR on prognosis in elderly ACS patients.

Recently, renal hyperfiltration has also shown predictive value for adverse clinical outcomes in several studies, which might be related to the activation of the renin-angiotensin-aldosterone system and the increased activity of the sympathetic nervous system [27]. Patients with type 2 diabetes and an eGFR <45 or ≥120 have an elevated risk of all-cause mortality [28]. In a study involving 8794 participants, in which low eGFR, normal eGFR, and high eGFR were defined by the 5th and 95th percentile, researchers reported that the low eGFR and the high eGFR group had a 2-times and 1.5-times increased risk of cardiovascular events compared with the normal eGFR group. These findings support a U-shaped relationship between eGFR and poor clinical outcomes [29]. Renal hyperfiltration has usually been considered a characteristic in the early stage of diabetes [27]. However, a study based on KIHD research data that was conducted to investigate the role of renal hyperfiltration in populations without diabetes, suggested that the correlation between renal hyperfiltration and mortality was not mediated by diabetes [30].

In our study, the L-eGFR and H-eGFR groups were defined by the 20th and 80th percentiles of the eGFR levels. Contrary to prior reports, the H-eGFR group which represented patients with renal hyperfiltration, did not show the same effect. Instead, our data revealed an association between H-eGFR and a reduced risk of MACE in the very elderly ACS patients. This seemingly paradoxical finding could be explained by several factors specific to our study population. First, the eGFR calculated by MDRD equation depended on serum creatinine concentration, which might be lower due to the decrease in muscle mass in the elderly [31]. Considering that the muscle mass of males is generally higher than that of females, there is reason to doubt that patients in the M-eGFR and H-eGFR groups, whose proportion of males was higher than that of the L-eGFR group, had better overall condition and lower risk of frailty. Additionally, given the physiological decline in renal function associated with aging, the cut-off for ‘high’ eGFR in our study may merely represent the upper end of the normal range for younger populations, rather than a truly pathological hyperfiltration state. Furthermore, our study specifically focused on a very elderly ACS population, in which survival bias is likely to be a significant factor. The higher eGFR may not represent a pathological state but rather indicate greater renal functional reserve. Lastly, the follow-up time of this study was considerably longer. For example, the hypertension in the very elderly (HYVET) trial reported a U-shaped relationship between eGFR and later cardiovascular events and mortality, which followed participants for a mean of only 2.1 years [32]. Our longer follow-up may have been necessary to uncover the distinct long-term prognostic role of high eGFR in this population. Therefore, the relationship between renal hyperfiltration and prognosis of advanced-age ACS patients remains unclear and further investigations into the underlying mechanisms are needed.

Our study has several limitations. First, as a single-center, retrospective observational study conducted in China with a relatively small sample size, our findings may lack generalizability to broader elderly ACS populations, and causality between eGFR and MACE cannot be inferred. Second, the lack of dynamic eGFR monitoring during follow-up is a constraint. Relying solely on baseline eGFR measurements to predict long-term prognosis is inherently limited. Third, commonly used cardiovascular medications, such as antiplatelet agents, statins, and renin-angiotensin-aldosterone system inhibitors, can influence both renal function and cardiovascular outcomes. However, accurate data on the use of medications were unavailable, as patients or family members could not provide specific details concerning their treatment. Fourth, while malnutrition is highly prevalent in the very oldest, our assessment was restricted to BMI and albumin levels, lacking more comprehensive nutritional indicators. Finally, the MDRD formula calculated eGFR based on serum creatinine, which is influenced by muscle mass and dietary protein intake in the elderly. Moreover, since the MDRD formula was primarily derived from populations with a mean age under 70 years, its application in our study cohort may have systematically underestimated the true glomerular filtration rate.

## 5. Conclusions

Reduced eGFR levels were independently associated with an increased risk for long-term MACE in advanced-age patients with ACS, implying that measurement of eGFR at admission may serve as a prognostic tool. Moreover, the H-eGFR group showed a better prognosis. Therefore, further investigations are warranted to investigate the underlying mechanism between renal hyperfiltration and prognosis in elderly ACS patients to help guide us to improve clinical outcomes through treatments targeting renal function.

## Availability of Data and Materials

The data that support the findings of this study are not openly available due to reasons of sensitivity and are available from the corresponding author upon reasonable request.

## Supplementary Material

Supplementary material associated with this article can be found, in the online version, at https://doi.org/10.31083/RCM45446.
