# Supplementary material for: Prognostic Value of Estimated Glomerular Filtration Rate in Older Patients With Acute Coronary Syndrome
Source: Rev Cardiovasc Med. 2026 Feb 11;27(2):45446. doi: 10.31083/RCM45446 (PMC12959998; doi:10.31083/RCM45446)
Supplement: Supplementary file 1 [file 2153-8174-27-2-45446-s1.zip › Supplementary Material.docx]

**Prognostic Value of Estimated Glomerular Filtration Rate in Elderly Patients with Acute Coronary Syndrome**

**Supplementary material**

| **Supplementary Table 1. Baseline characteristics of follow-up and lost population.** | | | |
| --- | --- | --- | --- |
| **Variables** | **Follow-up (N = 507)** | **Lost (N = 44)** | ***p* value** |
| **Male, n (%)** | 314 (61.9%) | 28 (63.6%) | 0.823 |
| **Age, years** | 81.00 (80.00, 83.00) | 81.00 (80.00, 83.00) | 0.891 |
| **BMI, kg/m2** | 24.4 (3.2) | 25.0 (3.7) | 0.474 |
| **Hypertension, n (%)** | 406 (80.1%) | 32 (72.7%) | 0.247 |
| **Dyslipidemia, n (%)** | 409 (80.7%) | 32 (72.7%) | 0.206 |
| **Diabetes, n (%)** | 179 (35.3%) | 15 (34.1%) | 0.871 |
| **Smoking, n (%)** | 205 (40.4%) | 24 (54.5%) | 0.068 |
| **Prior MI, n (%)** | 119 (23.5%) | 9 (20.5%) | 0.649 |
| **History of stroke/TIA, n (%)** | 132 (26.0%) | 8 (18.2%) | 0.251 |
| **Atrial fibrillation, n (%)** | 84 (16.6%) | 8 (18.2%) | 0.783 |
| **Classification of ACS** |  |  | 0.175 |
| **Unstable angina, n (%)** | 356 (70.2%) | 25 (56.8%) |  |
| **NSTEMI, n (%)** | 75 (14.8%) | 10 (22.7%) |  |
| **STEMI, n (%)** | 76 (15.0%) | 9 (20.5%) |  |
| **HR，bpm** | 67 (62, 75) | 73 (66, 82) | *0.011* |
| **SBP, mmHg** | 130 (120, 140) | 130 (119, 149) | 0.913 |
| **DBP, mmHg** | 70 (63, 80) | 70 (65, 80) | 0.148 |
| **Leukocyte, *10^9^/L** | 6.50 (5.51, 7.85) | 6.42 (4.97, 7.68) | 0.372 |
| **Albumin, g/L** | 39.4 (36.7, 42.5) | 38.9 (36.5, 42.1) | 0.679 |
| **TG, mmol/L** | 1.28 (0.94, 1.71) | 1.30 (1.00, 1.66) | 0.809 |
| **TC, mmol/L** | 3.81 (3.31, 4.41) | 4.06 (3.31, 4.70) | 0.244 |
| **HDL-C, mmol/L** | 1.08 (0.91, 1.31) | 1.10 (0.88, 1.26) | 0.534 |
| **LDL-C, mmol/L** | 2.22 (1.80, 2.65) | 2.37 (1.76, 3.11) | 0.227 |
| **eGFR, mL/min per 1.73m^2^** | 88.98 (24.36) | 88.99 (30.59) | 0.859 |
| **Uric acid, μmol/L** | 340.81 (279.08, 410.42) | 399.15 (312.92, 465.06) | *0.026* |
| **hsCRP, mg/L** | 2.37 (1.14, 7.24) | 2.59 (1.63, 8.30) | 0.199 |
| **HbA1c, %** | 6.2 (5.8, 6.8) | 6.2 (5.9, 6.9) | 0.947 |
| **NT-proBNP, pg/ml** | 680.2 (349.2, 1,205.7) | 863.4 (533.3, 2,011.7) | *0.016* |
| **LVEF, %** | 60.0 (56.0, 65.0) | 60.0 (54.5, 64.2) | 0.111 |
| Abbreviations: ACS, acute coronary syndrome; BMI, body mass index; DBP, diastolic blood pressure; eGFR, estimated glomerular filtration rate; HbA1c, hemoglobin A1c; HDL-C, high-density lipoprotein cholesterol; HR, heart rate; hsCRP, high-sensitivity C-reactive protein; LDL-C, low-density lipoprotein cholesterol; LVEF, left ventricular ejection fraction; MI, myocardial infarction; NSTEMI, non-ST-segment elevation myocardial infarction; NT-proBNP, N-terminal pro-brain natriuretic peptide; SBP, systolic blood pressure; STEMI, ST-segment elevation myocardial infarction; TC, total cholesterol; TG, triglyceride; TIA, transient ischemic attack. | | | |

| **Supplementary Table 2. Baseline characteristics of follow-up population in non-MACE group and MACE group.** | | | | |
| --- | --- | --- | --- | --- |
| **Variables** | **Overall (N = 507)** | **Non-MACE group（N=260）** | **MACE group（N=247）** | ***p* value** |
| **Male, n (%)** | 314 (61.9%) | 158 (60.8%) | 156 (63.2%) | 0.580 |
| **Age, years** | 81.00 (80.00, 83.00) | 81.00 (80.00, 83.00) | 81.00 (80.00, 83.00) | 0.085 |
| **BMI, kg/m^2^** | 24.4 (3.2) | 24.2 (3.1) | 24.5 (3.3) | 0.405 |
| **Hypertension, n (%)** | 406 (80.1%) | 203 (78.1%) | 203 (82.2%) | 0.247 |
| **Dyslipidemia, n (%)** | 409 (80.7%) | 204 (78.5%) | 205 (83.0%) | 0.196 |
| **Diabetes, n (%)** | 179 (35.3%) | 82 (31.5%) | 97 (39.3%) | 0.069 |
| **Smoking, n (%)** | 205 (40.4%) | 94 (36.2%) | 111 (44.9%) | *0.044* |
| **Prior MI, n (%)** | 119 (23.5%) | 51 (19.6%) | 68 (27.5%) | *0.036* |
| **History of stroke/TIA, n (%)** | 132 (26.0%) | 56 (21.5%) | 76 (30.8%) | *0.018* |
| **Atrial fibrillation, n (%)** | 84 (16.6%) | 31 (11.9%) | 53 (21.5%) | *0.004* |
| **Classification of ACS** |  |  |  | 0.644 |
| **Unstable angina, n (%)** | 356 (70.2%) | 184 (70.8%) | 172 (69.6%) |  |
| **NSTEMI, n (%)** | 75 (14.8%) | 35 (13.5%) | 40 (16.2%) |  |
| **STEMI, n (%)** | 76 (15.0%) | 41 (15.8%) | 35 (14.2%) |  |
| **HR，bpm** | 67 (62, 75) | 67 (61, 74) | 67 (62, 75) | 0.582 |
| **SBP, mmHg** | 130 (120, 140) | 130 (120, 140) | 130 (120, 145) | 0.182 |
| **DBP, mmHg** | 70 (63, 80) | 70 (62, 80) | 70 (63, 80) | 0.856 |
| **Leukocyte, *10^9^/L** | 6.50 (5.51, 7.85) | 6.43 (5.49, 7.74) | 6.57 (5.53, 8.05) | 0.436 |
| **Albumin, g/L** | 39.4 (36.7, 42.5) | 39.4 (36.9, 42.9) | 39.4 (36.2, 42.3) | 0.244 |
| **TG, mmol/L** | 1.28 (0.94, 1.71) | 1.30 (0.93, 1.70) | 1.26 (0.98, 1.72) | 0.511 |
| **TC, mmol/L** | 3.81 (3.31, 4.41) | 3.80 (3.39, 4.36) | 3.83 (3.27, 4.47) | 0.783 |
| **HDL-C, mmol/L** | 1.08 (0.91, 1.31) | 1.10 (0.92, 1.30) | 1.06 (0.89, 1.31) | 0.304 |
| **LDL-C, mmol/L** | 2.22 (1.80, 2.65) | 2.17 (1.85, 2.61) | 2.26 (1.72, 2.72) | 0.963 |
| **eGFR, mL/min per 1.73m^2^** | 88.98 (24.36) | 92.89 (24.13) | 84.87 (23.97) | *<0.001* |
| **Uric acid, μmol/L** | 340.81 (279.08, 410.42) | 328.23 (269.65, 388.96) | 356.88 (289.48, 433.25) | *<0.001* |
| **hsCRP, mg/L** | 2.37 (1.14, 7.24) | 2.01 (1.03, 5.24) | 2.61 (1.31, 10.47) | *0.007* |
| **HbA1c, %** | 6.2 (5.8, 6.8) | 6.2 (5.8, 6.7) | 6.3 (5.9, 7.0) | 0.287 |
| **NT-proBNP, pg/ml** | 680.2 (349.2, 1,205.7) | 540.5 (243.1, 837.3) | 875.7 (477.8, 1,511.6) | *<0.001* |
| **LVEF, %** | 60.0 (56.0, 65.0) | 62.0 (58.0, 67.0) | 60.0 (55.0, 63.0) | *<0.001* |
| Abbreviations: ACS, acute coronary syndrome; BMI, body mass index; DBP, diastolic blood pressure; eGFR, estimated glomerular filtration rate; HbA1c, hemoglobin A1c; HDL-C, high-density lipoprotein cholesterol; HR, heart rate; hsCRP, high-sensitivity C-reactive protein; LDL-C, low-density lipoprotein cholesterol; LVEF, left ventricular ejection fraction; MACE, major adverse cardiovascular events; MI, myocardial infarction; NSTEMI, non-ST-segment elevation myocardial infarction; NT-proBNP, N-terminal pro-brain natriuretic peptide; SBP, systolic blood pressure; STEMI, ST-segment elevation myocardial infarction; TC, total cholesterol; TG, triglyceride; TIA, transient ischemic attack. | | | | |

| **Supplementary Table 3. Predictive value of eGFR tertiles groups.** | | | |
| --- | --- | --- | --- |
| **Variables** | **HR** | **95%CI** | ***p* value** |
|  | **Univariate** | | |
| **T2 group** | Reference |  |  |
| **T1 group** | 1.591 | （1.179-2.147） | *0.002* |
| **T3 group** | 0.869 | （0.629-1.200） | 0.394 |
|  | **Multivariate** | | |
| **Model 1** |  |  |  |
| **T2 group** | Reference |  |  |
| **T1 group** | 1.657 | （1.216-2.258） | *0.001* |
| **T3 group** | 0.927 | （0.666-1.290） | 0.651 |
| **Model 2** |  |  |  |
| **T2 group** | Reference |  |  |
| **T1 group** | 1.457 | (1.052-2.016) | *0.023* |
| **T3 group** | 0.984 | (0.702-1.381) | 0.927 |
| Model 1: adjusted for age, gender and BMI; Model 2: adjusted for model 1 plus albumin and uric acid. | | | |

| **Supplementary Table 4. Predictive value of eGFR groups according to CKD staging criteria.** | | | |
| --- | --- | --- | --- |
| **Variables** | **HR** | **95%CI** | ***p* value** |
|  | **Univariate** | | |
| **group 2** | Reference |  |  |
| **group 1** | 1.521 | （1.037-2.231） | *0.032* |
| **group 3** | 0.677 | （0.517-0.887） | *0.005* |
|  | **Multivariate** | | |
| **Model 1** |  |  |  |
| **group 2** | Reference |  |  |
| **group 1** | 1.521 | （1.021-2.263） | *0.039* |
| **group 3** | 0.683 | （0.519-0.899） | *0.007* |
| **Model 2** |  |  |  |
| **group 2** | Reference |  |  |
| **group 1** | 1.254 | (0.810-1.943) | 0.311 |
| **group 3** | 0.743 | (0.557-0.990) | *0.042* |
| Model 1: adjusted for age, gender and BMI; Model 2: adjusted for model 1 plus albumin and uric acid. | | | |

**
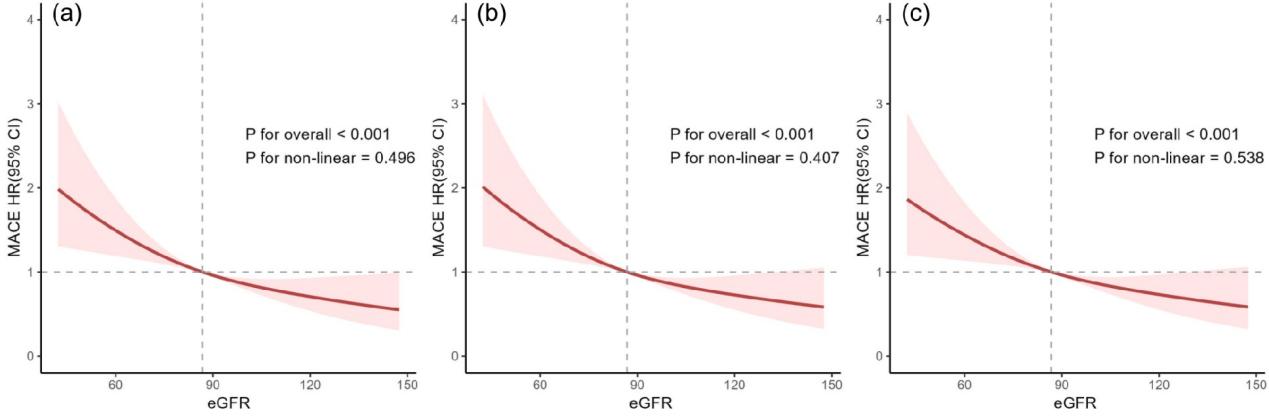
**

**Supplementary Fig. 1. Restricted cubic splines of the association between eGFR and MACE.** (a) included eGFR only; (b) adjusted for age, gender and BMI; (c) adjusted for (b) plus hypertension, dyslipidemia, diabetes, smoking, and hsCRP. eGFR, estimated glomerular filtration rate; MACE, major adverse cardiovascular events.


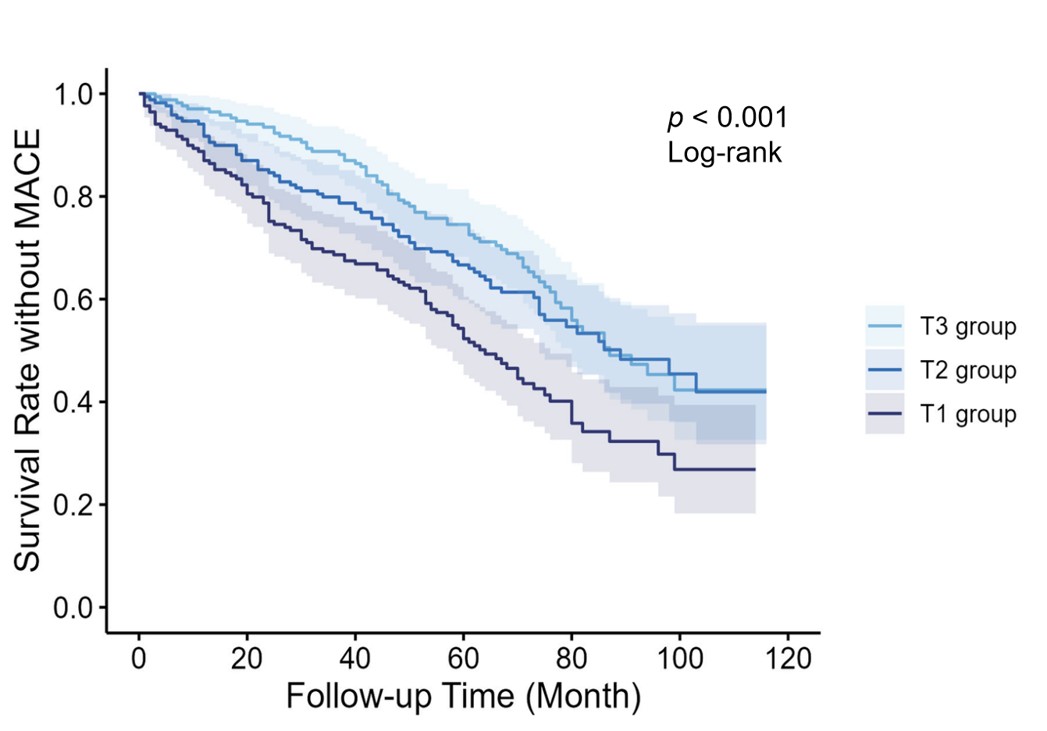


**Supplementary Fig. 2. Kaplan Meier survival curves for eGFR tertiles groups.** eGFR, estimated glomerular filtration rate; MACE, major adverse cardiovascular events.


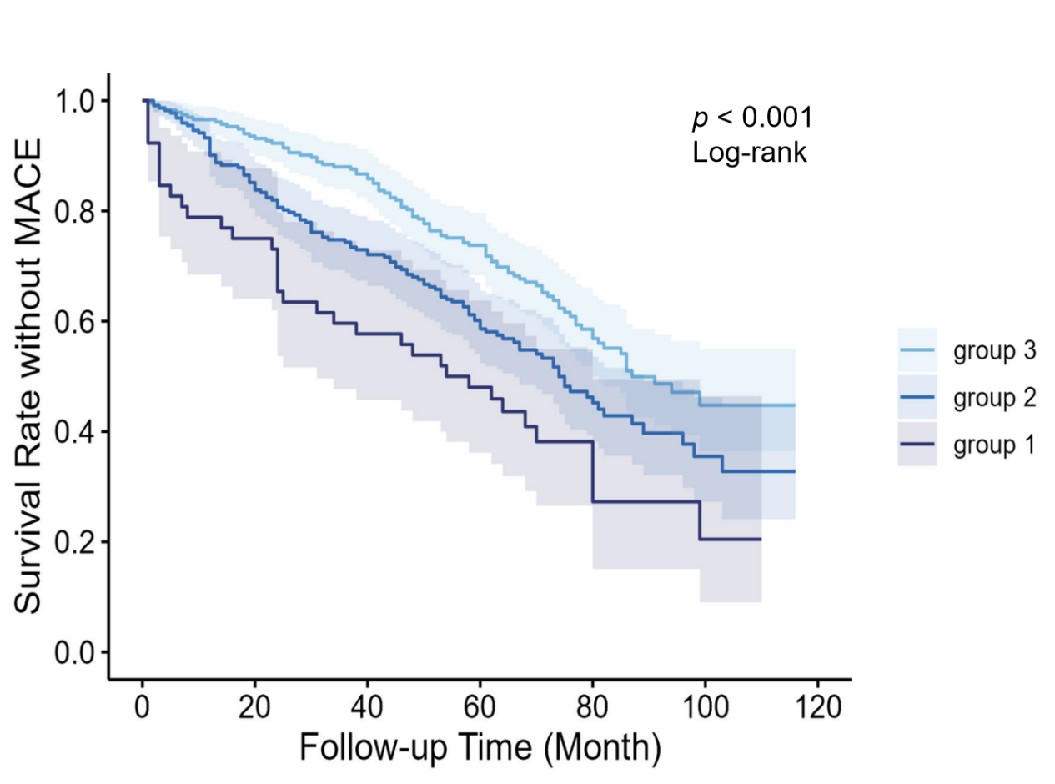


**Supplementary Fig. 3. Kaplan Meier survival curves for eGFR groups according to CKD staging criteria.** CKD, chronic kidney disease; eGFR, estimated glomerular filtration rate; MACE, major adverse cardiovascular events.
